# Poetry as reflexive lens: humanising nursing practice in robotic-assisted surgery

**DOI:** 10.1177/17449871251407831

**Published:** 2026-01-20

**Authors:** Lian Lee

**Affiliations:** Oxford Brookes University, UK

My research examined nursing practice within the highly technological environment of robotic-assisted surgery, where nurses navigate the intersection of human expertise and advanced digital systems. In this setting, nurses occupy a pivotal role as the constant presence at the patient’s side, overseeing safety, coordinating communication, and supporting surgeons who are physically distant from the operating table. Communication in surgery is integral to the fundamentals of perioperative nursing practice and patient safety. Research exploring team communication in robotic-assisted surgery (RAS) is evident in the literature, but little attention has been focused on how the experiences of operating room nurses’ communication affect safety, practice and patient care outcomes (Lee et al., 2024). My doctoral study illuminated the often-unseen practices that nurses employ to maintain safety: vigilance, situational awareness, anticipatory action, and a willingness to speak up when patient well-being is at risk. These practices, though sometimes enacted in silence, represent sophisticated clinical judgement and the essence of perioperative nursing. Through examining nurses’ experiences, the research revealed how humanism, intuition, and professional identity persist and evolve within a technologically mediated surgical environment. To capture this often-invisible expertise, I transformed the participants’ shared experiences into poetic form. The following poem was crafted as part of the findings, giving voice to the emotional resonance embedded in robotic-assisted surgical nursing:

*Nurses’ stories gathered*,

*Intense emotions unravel*,

*Intelligent minds*,

*Robotics expert's hands*,

*Artfully, they choreograph*,

*Quietly, they control*,

*Gracefully they serve*,

*Unspoken intelligence*,

*Silence is golden*,

*Magnificent partnership, they transformed*,

*Robot and nurses*,

*Eloquently, they dance*,

*Passionate, poignant expressions*,

*Vividly unfolding*,

*Messages ignite in darkness*,

*Juxtaposition they conquered*,

*Mastery of trust, nursing science and art.*

The poem reflects the harmonious interplay between human skill and technological complexity – a choreography in which nurses’ vigilance, compassion, and professionalism are central to safe surgical care.

## Reflexivity: Introducing nuance and the human factor

A reflexive thematic analysis (RTA) approach was central to this study. RTA asserts that reality and meaning are constructed through social and cultural processes, shaped by our interactions and contexts (Braun and Clarke, 2023). This approach allows me to acknowledge how my dual position as practitioner and researcher shaped the research process, as well as the art of nursing through a humanistic approach within the highly scientific/robotic surgical environment (Henry, 2018). Reflexivity enhances researchers’ engagement with the data and participants as described by Heidegger’s philosophy of ‘Being’ Heidegger (1927/2008, 1943/2008). The choices we make through reflexivity helps gaining meaning through our relationships in the moment, which in this instance during my researcher’s reflexivity diary and synthesis of the findings from participants’ stories had allowed me to remain present and ‘being’ in those moments. This unique relationship is a continuum which forms part of the methodology unique in qualitative study. The findings in my study, supported by both literature and participant narratives, indicate that advanced technology can diminish the humanistic aspect of care, widening the gap between patients and robotic surgery teams. Holistic nursing in this field therefore requires deliberate reflection – a critical examination of practice – to achieve a meaningful understanding of the context that shapes our decisions and thinking, as advocated by Heidegger (1927/2008, 1943/2008). This philosophical lens enriches the researcher’s reflective process, and poetry writing is a craft that serves this process through intentionally generating creative thinking and mindful spaces. In the context of RAS, nurses are required to be present to overcome multiple disruptions which are distraction to their care delivery. This presence that I developed through my nursing experiences in practice helped me to embrace this unique perspective of poetry writing in my research journey.

Reflexivity created a space in which I could engage deeply with the participants’ stories, questioning assumptions, exploring moments of discomfort, and recognising how my own nursing experiences informed interpretation. This reflexive stance brought nuance to the analysis, ensuring that the complexity of nurses’ voices was neither simplified nor overshadowed by the technological setting. The interview encounters became relational spaces where meaning was shared, co-constructed and understood, reflecting the profound human dimension that underpins clinical practice. Subsequently, the reflexive art of poetry writing enabled a dwelling with these accounts and avoiding a rush to data reduction. Reflexivity therefore operated as both a methodological tool and a professional act of authenticity, giving due space to participant accounts and affirming that nursing research is inseparable from the humanity of those who conduct and participate in it.

## Poetry and its role in the reflexive process

Crafting poems became an integral part of my reflexive practice, offering an expressive form through which I could engage with the emotional texture of the data. William Blake (2019) defines poetry as the art of capturing the infinite in the smallest moments. In research, the crafting of poems from the data both gives space for reflexivity and is also a means by which to distil complex experiences, whilst revealing the subtleties of thought, feeling, and interaction that might otherwise remain hidden beneath conventional academic syntax (Hopkinson, 2015; Tarbi and Morgan, 2022). Within this study, poetry enabled me to honour the participants’ narratives by capturing the rhythm, intensity, and humanity of their experiences. Writing poems during analysis allowed me to pause, reflect, and make sense of the liminal ‘third space’ between researcher and participants – a space where empathy, vulnerability, and insight coexist. Rather than detracting from scientific rigour, poetry enriched it, providing an avenue for emotionally attuned interpretation. Using poetry alongside research provides a humanistic, empathetic lens on experience, inviting reflection on the emotional, relational, and existential dimensions of caregiving beyond clinical data (Madden and Bowers, 2025). Poetry acted as a bridge between art and evidence, supporting a reflexive synthesis that respected both the precision of empirical analysis and the lived realities of nursing practice.

As I synthesised the data, poetry allowed me to step into, and dwell in – for a while, a contemplative space where analysis and emotion coexisted. The following poem, written during this phase of the study, captures the merging of scholarly inquiry with personal reflexivity, symbolising how lived experience evolves into research knowledge:

*A researcher’s mind and soul*

*She holds her beloved pen*

*She inks every line with deep emotions*

*Artfully and scientifically, she reveals*

*An innocent piece of a profound story*

*Eloquently she communicates*

*A transformative journey*

*Lived experiences become science*

## Methodological insights: The art and science of nursing and nursing research

From a methodological perspective, this study demonstrated that nursing research, much like nursing practice, involves a dynamic interplay between art and science. The scientific dimensions: systematic inquiry, rigorous analysis and philosophical grounding in constructivism provided a structure for understanding nurses’ experiences within RAS. Constructivism is a philosophy that asserts knowledge is not a passive entity to be received; instead, it is actively created by individuals through their experiences and interactions (Guba and Lincoln, 1994). Yet, the artistry of nursing, and in this case nursing research, was equally essential, shaping the ability to interpret narratives, recognise meaning and remain present with the emotional resonance of the data. Using poetry and reflexivity together revealed that knowledge in nursing is not solely produced through measurable outcomes, but through attentiveness, relationality, and compassionate understanding – and in addition, different ways of writing. This methodological fusion highlights how qualitative research can uncover layers of practice that are often overlooked in technologically driven environments, and further, in the research process. It affirms that, even in an era marked by robotics and Artifical Intelligence, the creative, responsive, humanistic foundations of nursing: compassion, empathy, intuition, moral agency – remain central to both clinical excellence and scholarly inquiry.
